# Primary Extraosseous Ewing’s Sarcoma of the Lung: Radiologic and Pathologic correlation

**DOI:** 10.7759/cureus.14830

**Published:** 2021-05-04

**Authors:** Saffet Öztürk, Esin Kurtulus Ozturk, Nilgun Isiksalan Ozbulbul, Berat Acu, Emine Dundar

**Affiliations:** 1 Radiology, Sungurlu State Hospital, Çorum, TUR; 2 Radiology, Kutahya University of Health Sciences Faculty of Medicine, Kutahya, TUR; 3 Radiology, Ankara City Hospital, Ankara, TUR; 4 Radiology, Osmangazi University Faculty of Medicine, Eskisehir, TUR; 5 Pathology, Osmangazi University Faculty of Medicine, Eskisehir, TUR

**Keywords:** ewing’s sarcoma, primary lung, ct, mri

## Abstract

Ewing’s sarcoma (ES) is a rare and highly aggressive tumor belonging to a family of neoplasms of neuroectodermal origin, which primarily affects the bones or soft tissues. ES originating from lung parenchyma without chest wall involvement is extremely rare with less than 40 cases reported in the English literature. A 41-year-old man admitted to the thoracic surgery department presenting with intermittent non-productive cough, dyspnea, left-sided chest pain for two months for further evaluation and treatment with a preliminary diagnosis of pulmonary mass. Contrast-enhanced thorax CT and MRI revealed a large heterogeneous soft-tissue mass in the left lower lobe with no distant metastases or occult primary tumor. Following the percutaneous transthoracic biopsy, histopathological and immunohistochemical results were consistent with primary pulmonary ES. Though rare, primary pulmonary ES should be considered in the differential diagnosis of young patients presenting with a large heterogeneous soft tissue mass in the lung. This case report highlights the diagnosis, radiologic and pathologic findings, and management of primary pulmonary ES.

## Introduction

The Ewing’s sarcoma family tumor (ESFT) are rare malignant tumors originating from neuroectodermal cells that commonly affect the long bones, such as arms, legs, ribs, vertebral column, and pelvis. ESFT occurs mainly in pediatric patients and young adults. Osseous Ewing’s sarcoma (ES), extraosseous ES, peripheral primitive neuroectodermal tumor (PNET), and malignant neuroectodermal tumor of the chest wall (Askin tumor) are all considered members of the ESFT. Approximately 16% of all ESFT are extraosseous [1-3]. Primary pulmonary ES is extremely rare and can be difficult to distinguish from the other lung tumors due to its similar presentation and nonspecific symptoms. The purpose of this report is to present a case of primary extraosseous ES of the lung presenting with a rapidly growing large heterogeneous soft tissue mass in light of the literature.

## Case presentation

**Figure 1:**
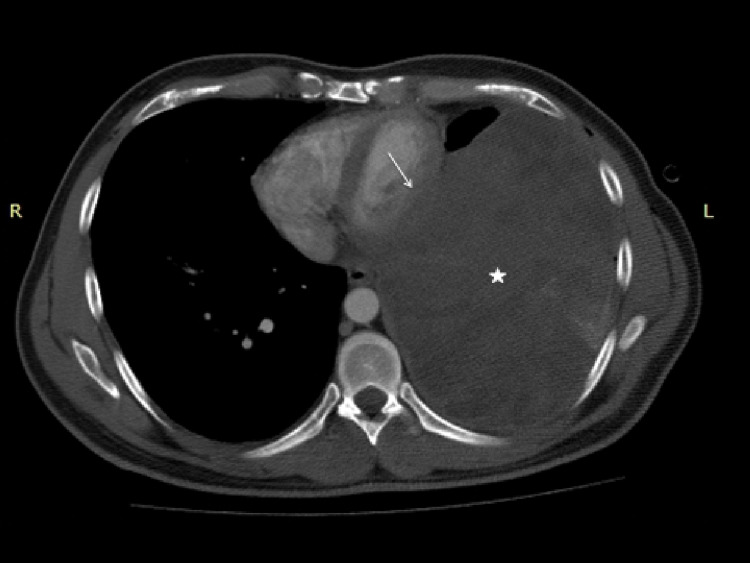
An axial contrast-enhanced chest computerized tomography image The image shows a large, relatively well-defined, noncalcified heterogenous soft tissue mass (star) within the left lower lobe that compressed and displaced the mediastinum and left ventricule (arrow).

A 41-year-old man admitted to thoracic surgery department with of intermittent non-productive cough, dyspnea and chest pain of two months. The past history was not contributory and there was no family history of any cancer. The laboratory tests, which included assessment of tumor markers (serum levels of ferritin, carcinoembryonic antigen [CEA], alpha-fetoprotein, carbohydrate antigens [CA 125, CA 15.3, CA 19.9]) were completely normal. Physical examination of the lungs revealed absent breath sounds at the left lung base. A chest radiograph confirmed a large pleural effusion and total atelectasis of the left lower lobe. A diagnostic thoracentesis of pleural effusion was performed that was cytologically negative. A chest tube was then inserted in the left side posterior pleural cavity. Due to continuity of the opacity on subsequent radiograph, the patient was investigated with contrast-enhanced chest multidetector computed tomography (CT) and contrast-enhanced chest magnetic resonance imaging (MRI) for suspicion of mass. CT scan was performed using 64-slice CT scanner (Aquilion; Toshiba Medical Systems, Otawara, Japan) after intravenous contrast administration (Omnipaque 350, Nycomed Amersham, Princeton, NJ, USA). And the patient was also scanned with 3T MRI scanner (GE Healthcare, Milwaukee, WI, USA) with intravenous gadolinium contrast administration (Dotarem, gadoterate meglumine; Guerbet, Aulnay-sous-bois, France). Contrast-enhanced CT and MRI revealed a large, relatively well-defined, noncalcified heterogenous soft tissue mass measuring approximately 15x15x14 cm within the left lower lobe that compressed and displaced the left hilum, mediastinum and left atrium and ventricule (Figure 1).

**Figure 2:**
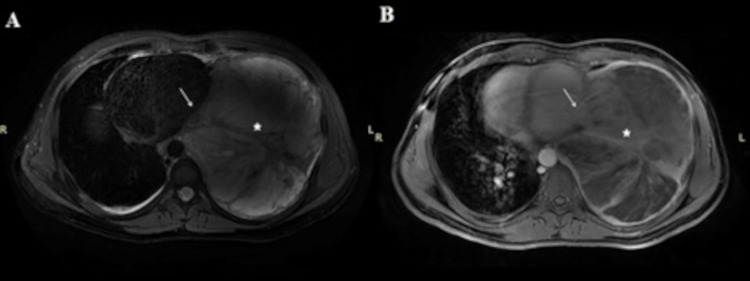
An axial fat-sat T2 (PROPELLER) image (A) and an axial contrast-enhanced fat-sat T1 image (B) The images demonstrate the large heterogenous soft tissue mass (star) within the left lower lobe that compressed and displaced the mediastinum and left ventricule (arrow). Also, the mediastinum was invaded by the tumor with an irregular margin (arrow).

On MRI, the mediastinum was invaded by the tumor with irregular margin (Figure 2).

**Figure 3:**
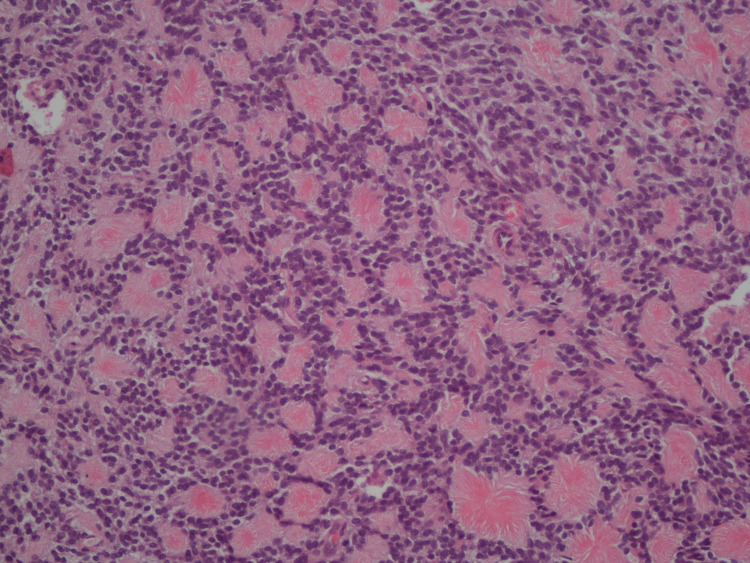
Ewing’s sarcoma showing uniform small cells with round nuclei and fine chromatin. Homer Wright rosettes are not seen (hematoxylin and eosin, 200x)

Also 18 F-FDG PET/CT scan confirmed a metabolically active mass (SUVmax = 30.3) in the left lower lobe parenchyma, with no additional increased activity to suggest an occult primary or metastatic disease. Ultrasound-guided tru-cut needle lung biopsy was performed. Histologically, the tumor was composed of uniform small round cells in sheets with scant clear cytoplasm and round nuclei containing fine chromatin (Figure 3).

**Figure 4:**
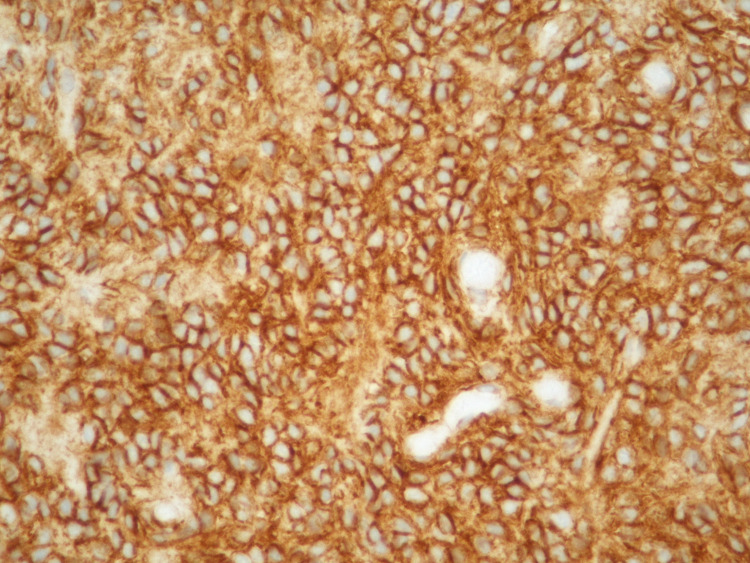
Diffuse membranous staining of Ewing’s sarcoma for CD99 (immunohistochemistry, 200x)

Immunohistochemical stain results of the tumor cells were positive for smooth muscle actin, vimentin, neuron-specific enolase, synaptophysin and the tumor showed similar strong reactivity for CD99 (Figure 4).

The tumor did not express cytokeratin, carcinoembryogenic antigen, desmin, and protein S-100. All other antibodies were negative. Information about molecular testing translocation/FISH studies could not be found in our data system. These findings confirmed the diagnosis of ESFT. The patient was referred to the medical oncology department for palliative chemotherapy regimen consisting of cyclophosphamide, doxorubicin, ifosfamide, and etoposide. Written informed consent was obtained from the patient.

## Discussion

Extraosseous ES is extremely rare originating from lung parenchyma that has been reported in various organ including pancreas, kidney, ovary, palate and myocardium [2,3]. Despite a variety of locations, ESFT shares a common histological and morphological feature composed of closely clustered small primitive round cells [4]. Primary pulmonary ES without chest wall involvement has been reported with approximately 40 cases in the English literature. In these cases, most patients are adolescents or young adults with a mean age of 28 years (range 8-67 years), slightly older than in osseous ES with a mean age of 20 years [5]. Clinical features at presentation vary depending on tumor size and location. Blood or biochemical tests are nonspecific and inadequate to obtain an accurate diagnosis. Nonspecific signs of tumor, such as an elevated sedimentation rate, moderate anemia, or leukocytosis, may be noted [6].

Extraosseous ES most often presents as a rapidly growing painless solitary mass in soft tissues, generally manifesting up to 20 cm at the initial presentation. This course of tumor might be responsible for delaying diagnosis and treatment because increased primary tumor volume correlates with overall survival [7]. Imaging plays an essential role in detection, diagnosis, staging, treatment and surveillance. On CT, pulmonary ES similarly show heterogeneous enhancement with hypo or hyperdense foci due to necrosis or hemorrhage. Calcification is atypical, occurring in approximately 10% of these tumors at presentation. Despite their size, pulmonary ES most often tend to be unilateral. Pleural thickening and malignant effusion can be seen. Lymphadenopathy indicating lymph node metastasis is rare, reported in 0%-12% of cases. Pulmonary ES can invade or displace the mediastinum and mediastinal organs. Therefore, MRI has a potential role in evaluating adjacent organ invasion and locoregional staging of tumors [2,7]. In the diagnosis of ES, an FDG PET scan does not improve diagnostic accuracy, but its sensitivity and specificity are quite high, 96% and 78%, respectively [8].

Primary or metastatic pulmonary tumors cannot be confidently differentiated from ES by imaging. As a rule, exact diagnosis is based on the histopathology and the immunohistology of the specimen taken by biopsy or surgery for general diagnosis of ESFT [7]. Evaluation combining light microscopy, immunohistochemistry, molecular pathology, and/or genetics is often needed to accurately diagnose and differentiate from small-cell carcinoma, adenocarcinoma, squamous cell carcinoma, melanoma, rhabdomyosarcoma, and lymphoma. All these tumors composed of closely packed in sheets of small round blue cells with scant eosinophilic cytoplasm have similar appearance by light microscopy. It is not easy to distinguish Ewing’s sarcoma from these lesions by focusing only on histological studies. Therefore, immunohistochemical staining may aid in providing a more accurate diagnosis. Strong staining for glycoprotein p30/32 (CD99), the product of MIC2 gene, has recently been used in the evaluation of the ESFT [9]. In addition, ESFT includes reciprocal translocations, of which approximately 90% occur between the long arm of chromosomes 11 and 22, t(11;22)(q24;q12), resulting in the formation of aberrant hybrid proteins generated by a fusion of the EWS and FLI-1 genes. The presence of these fusion genes might be the defining criterion for the ESFTs [10].

Given the rarity of primary pulmonary ES, diagnosis and therapeutic management can be challenging. The treatment includes various combinations of surgery, chemotherapy (multiagent chemotherapy regimen) and radiation therapy. Adjuvant and/or neoadjuvant chemotherapy may affect the overall survival and improve the treatment results in the future. Some studies have been reported that ESFT can respond dramatically to initial therapy, with robust initial responses predicting a better outcome [4,7].

## Conclusions

Despite being a rarity, pulmonary ES should always be considered in the differential diagnosis in young patients with pulmonary soft tissue tumors. Diagnostic imaging has an increasingly important role in accurate diagnosis, management and follow-up. The definitive diagnosis of ESFT is made based on histomorphologic immunohistochemical and molecular genetic features.

## References

[1] Hwang SK, Kim DK, Park SI, Kim YH, Kim HR (2014). Primary Ewing's sarcoma of the lung. Korean J Thorac Cardiovasc Surg.

[2] Lee YY, Kim DH, Lee JH (2007). Primary pulmonary Ewing's sarcoma/primitive neuroectodermal tumor in a 67-year-old man. J Korean Med Sci.

[3] Suárez Antelo J, Rodríguez García C, Montero Martínez C, Verea Hernando H (2010). [Pulmonary Ewing sarcoma/primitive neuroectodermal tumor: a case report and a review of the literature] (Article in Spanish). Arch Bronconeumol.

[4] Takahashi D, Nagayama J, Nagatoshi Y (2007). Primary Ewing's sarcoma family tumors of the lung a case report and review of the literature. Jpn J Clin Oncol.

[5] Mizuguchi K, Minato H, Onishi H, Mitani Y, Kawai J (2016). Cytopathological findings of primary pulmonary Ewing family of tumors with EWSR1 translocation: a case report. Thorac Cancer.

[6] Bernstein M, Kovar H, Paulussen M, Randall RL, Schuck A, Teot LA, Juergens H (2006). Ewing's sarcoma family of tumors: current management. Oncologist.

[7] Javery O, Krajewski K, O'Regan K, Kis B, Giardino A, Jagannathan J, Ramaiya NH (2011). A to Z of extraskeletal Ewing sarcoma family of tumors in adults: imaging features of primary disease, metastatic patterns, and treatment responses. AJR Am J Roentgenol.

[8] Györke T, Zajic T, Lange A, Schäfer O, Moser E, Makó E, Brink I (2006). Impact of FDG PET for staging of Ewing sarcomas and primitive neuroectodermal tumours. Nucl Med Commun.

[9] Fellinger EJ, Garin-Chesa P, Triche TJ, Huvos AG, Rettig WJ (1991). Immunohistochemical analysis of Ewing's sarcoma cell surface antigen p30/32MIC2. Am J Pathol.

[10] Delattre O, Zucman J, Melot T (1994). The Ewing family of tumors--a subgroup of small-round-cell tumors defined by specific chimeric transcripts. N Engl J Med.

